# Parliament and Publications

**Published:** 1911-02-25

**Authors:** 


### Parliament and Publications.

### THE SALE OF SECRET REMEDIES.

Mr. Stephen Collins has given notice to ask the Home Secretary whether he is prepared to appoint a Select Committee to consider the advisability of issuing regulations making it compulsory upon all manufacturers of medicines liable to patent medicine duty to print on the label of the bottle or other vessel in which such is sold the full ingredients.

### THE MIDWIVES ROLL.

On March 31, 1910, the names on the Midwives Roll amounted to 29,209, an increase for the year of 1,928. Of the total 8,147 have passed the examination of the Central Midwives Board, and 9,643 have been admitted to the roll in virtue of prior certification. The total number of trained midwives is therefore 17,790, as against 11,419 untrained, the percentage being 61 and 39. Owing to the incompleteness of the returns made by the local supervising authorities, it is impossible to estimate with accuracy the respective proportions in the case of practising midwives. A large percentage of the trained women obtained their certificate without any intention of ever practising, many others have ceased to do so, and a considerable number practise in the Colonies or in foreign countries. There can be no doubt that at the present time the untrained practising midwives are largely in excess of the trained. During the year ended March 31, 1910, 53 penal cases were heard by the Board, with the result that 23 women were struck off the Roll, 14 censured, 13 cautioned, and 3 permitted to resign voluntarily.—"Report on the Work of the Central Midwives Board," Cd. 5505, price ½d.

### THE HOSPITALS OF BRITISH EAST AFRICA.

The two European hospitals in East Africa Protectorate admitted during the year 1909-10 a total of 200 cases, 89 of which were treated at Mombasa and 111 at Nairobi. At each of the headquarters stations of the provinces there is a native civil hospital in the charge of a medical officer, or in two instances of an assistant surgeon. In addition, there are some 20 civil dispensaries, to most of which is attached a small hospital. The country was visited, for the first time for ten years, by an outbreak of small-pox. Its spread was controlled by vigorous vaccination and altogether 59,374 vaccinations were performed. The lymph used was manufactured in the Nairobi laboratory, and, of the cases that could be followed, gave an average success of perfect vesicles in 95 per cent. The epidemic of sleeping sickness, which is confined to one small district in South Kavirondo, is declining.—Colonial Reports, No. 669, price 10d.

### THE HOSPITALS OF UGANDA.

The report of the Principal Medical Officer states that there was an increase in the number of admissions of natives to Government hospitals in Uganda during the year 1909. Natives are acquiring greater confidence in European methods of treatment. On the whole, the results of the preventive measures in the Uganda Protectorate against sleeping sickness must be regarded as highly satisfactory, and it is believed that the disease is beginning to show a decrease in Unyoro and Busogo. The process of removal of natives from contact with fly areas is being steadily extended.—Colonial Reports, No. 670, price 4½d.

### THE HOSPITALS OF MAURITIUS.

In Mauritius there are 13 hospitals and 28 dispensaries. The number of admissions to all hospitals was 23,205 in 1909, and the case mortality was 5.83 per cent. The number of cases treated at the dispensaries was 69,593. The number of children presented for vaccination at the public vaccine stations was 9,709, of whom 9,587 were successfully vaccinated.—Colonial Reports, No. 666, price 3½d.

### THE HEALTH OF BRITISH GUIANA.

In British Guiana there are five public hospitals in the principal centres of population, and the outlying districts are served by dispensary hospitals and dispensaries. The total expenditure on the Medical Department of the Colony during the year 1909-10, including quarantine services and vaccination, was £77,120 16s. 8d. of which £35,355 was for salaries; 16,889 patients were admitted to the hospitals during the year, and outdoor treatment was given to 74,646 persons. At the leper asylum Professor Deycke's Nastin treatment was continued under the supervision of the bacteriologist. The Surgeon-General states that it is as yet too early to make a definite pronouncement as to whether Nastin is a cure for leprosy, but in some cases there has undoubtedly been considerable improvement. The principal causes of death during the year were malarial fevers, diarrhœal diseases, phthisis and other forms of tuberculosis. Malarial fever continues to claim a large number of victims, and efforts are being made by legislative and other action to control the mosquito pest.—Colonial Reports, No. [illegible]71, price 3½d.

